# Performance of Microbiome Sequence Inference Methods in Environments with Varying Biomass

**DOI:** 10.1128/mSystems.00163-18

**Published:** 2019-02-19

**Authors:** Vincent Caruso, Xubo Song, Mark Asquith, Lisa Karstens

**Affiliations:** a Division of Bioinformatics and Computational Biology, Oregon Health and Science University, Portland, Oregon, USA; b Center for Spoken Language Understanding, Oregon Health and Science University, Portland, Oregon, USA; c Division of Arthritis and Rheumatic Diseases, Oregon Health and Science University, Portland, Oregon, USA; d Division of Urogynecology, Oregon Health and Science University, Portland, Oregon, USA; Institute for Systems Biology

**Keywords:** ASV methods, OTU clustering, bioinformatics, microbiome

## Abstract

Microbiome community composition plays an important role in human health, and while most research to date has focused on high-microbial-biomass communities, low-biomass communities are also important. However, contamination and technical noise make determining the true community signal difficult when biomass levels are low, and the influence of varying biomass on sequence processing methods has received little attention. Here, we benchmarked six methods that infer community composition from 16S rRNA sequence reads, using samples of varying biomass. We included two operational taxonomic unit (OTU) clustering algorithms, one entropy-based method, and three more-recent amplicon sequence variant (ASV) methods. We first compared inference results from high-biomass mock communities to assess baseline performance. We then benchmarked the methods on a dilution series made from a single mock community—samples that varied only in biomass. ASVs/OTUs inferred by each method were classified as representing expected community, technical noise, or contamination. With the high-biomass data, we found that the ASV methods had good sensitivity and precision, whereas the other methods suffered in one area or in both. Inferred contamination was present only in small proportions. With the dilution series, contamination represented an increasing proportion of the data from the inferred communities, regardless of the inference method used. However, correlation between inferred contaminants and sample biomass was strongest for the ASV methods and weakest for the OTU methods. Thus, no inference method on its own can distinguish true community sequences from contaminant sequences, but ASV methods provide the most accurate characterization of community and contaminants.

**IMPORTANCE** Microbial communities have important ramifications for human health, but determining their impact requires accurate characterization. Current technology makes microbiome sequence data more accessible than ever. However, popular software methods for analyzing these data are based on algorithms developed alongside older sequencing technology and smaller data sets and thus may not be adequate for modern, high-throughput data sets. Additionally, samples from environments where microbes are scarce present additional challenges to community characterization relative to high-biomass environments, an issue that is often ignored. We found that a new class of microbiome sequence processing tools, called amplicon sequence variant (ASV) methods, outperformed conventional methods. In samples representing low-biomass communities, where sample contamination becomes a significant confounding factor, the improved accuracy of ASV methods may allow more-robust computational identification of contaminants.

## Abstract (precis)

Microbial communities have important ramifications for human health, but determining their impact requires accurate characterization. Current technology makes microbiome sequence data more accessible than ever. However, popular software methods for analyzing these data are based on algorithms developed alongside older sequencing technology and smaller data sets and thus may not be adequate for modern, high-throughput data sets. Additionally, samples from environments where microbes are scarce present additional challenges to community characterization relative to high-biomass environments, an issue that is often ignored. We found that a new class of microbiome sequence processing tools, called amplicon sequence variant (ASV) methods, outperformed conventional methods. In samples representing low-biomass communities, where sample contamination becomes a significant confounding factor, the improved accuracy of ASV methods may allow more-robust computational identification of contaminants.

## INTRODUCTION

Microbiome research has established the crucial role of microbial communities in many environments, including the important link between human microbial communities at various body sites and a number of disorders, ranging from obesity (1) to irritable bowel disease (2) to Parkinson’s disease (3). While the majority of research has focused on environments with relatively high microbial biomass, such as the human gut, microbial communities are also found at much lower abundance in a variety of other environments. Some examples of low-biomass microbiomes are the urinary tract (4), mucosae of the lungs (5), and blood (6), as well as the built environment (7), including hospitals (8) and spacecraft assembly facilities (9). As with higher-biomass microbiomes, dysbioses of low-biomass microbiomes are also associated with disease, including urgency urinary incontinence (10, 11), cystic fibrosis, and asthma (12). Thus, these low-biomass environments are medically important.

Currently, a common method for profiling microbial communities is to sequence the 16S rRNA gene. Found in all prokaryotes, the 16S rRNA gene consists of hypervariable regions, which serve as barcodes to identify distinct organisms, flanked by highly conserved regions that offer a target for PCR primers to isolate and amplify the region of interest in a wide range of organisms. DNA sequencing reads generated from the 16S region are processed to remove sequencing noise and intraorganism variation, as well as to remove PCR chimeras. Clustering reads into operational taxonomic units (OTUs) has been the *de facto* standard for sequence inference with 16S rRNA gene sequencing data since at least 2006 (13). With OTU methods, the researcher selects a radius of variability (typically 3%), within which sequence differences are assumed to be due to variation within the taxonomic group or to random sequencer noise. All sequence reads within the chosen radius are clustered into a single OTU, representing one unit of analysis.

Recently, several methods have been published that take a different approach (14–16). These algorithms, which we (and others [17, 18]) refer to as amplicon sequence variant (ASV) methods, attempt to model the error of the sequencer and to cluster reads such that their distribution within clusters is consistent with the error model. This approach avoids making assumptions about the variation within a taxonomic group, a weakness of OTU methods (19). By considering both sequence similarity and abundance in the model, ASV methods account for the error profile that results from next-generation sequencing (NGS) experiments, which may produce tens of thousands of reads for a single 16S rRNA gene template sequence. Hence, ASV methods have the potential simultaneously to improve the sensitivity and specificity of 16S rRNA gene sequence inference compared to OTU methods.

Samples taken from an environment with low microbial biomass present distinct challenges (20, 21), and methods deemed appropriate for high-biomass samples—both in the laboratory and *in silico*—may not transfer well to low-biomass studies. In dealing with low-biomass samples, there is less starting template DNA for the PCR. Consequently, any contamination from extraction reagents or the laboratory environment makes up a larger fraction of the extracted sample than is the case with high-microbial-biomass samples (20). Additionally, the greater number of PCR cycles typically required with low-biomass samples may produce disproportionate quantities of contaminant sequences, depending on the amplification bias of the primers used (5). In other words, the sequencing of low-biomass microbiome communities suffers from a low signal-to-noise ratio, a problem not encountered in sequencing high-microbial-biomass communities, since contaminating sequences are overwhelmed by the community DNA of high-biomass samples.

In this study, we focused on *in silico* sequence inference and compared the performance characteristics of several inference methods to provide an unbiased assessment of performance in high-biomass settings, as well as to investigate how the starting DNA concentration of a sample affects the inferred community composition. To do this, we performed two distinct but related experiments. First, we compared selected methods applied to various mock community data sets to establish their performance on high-microbial-biomass samples of varying compositions. We then evaluated the same methods on a dilution series made from a single mock microbial community to see how inference results changed as the starting DNA concentration decreased. We hypothesized that ASV methods would be both more sensitive and specific than OTU methods, regardless of the starting biomass. We also anticipated that decreasing the starting DNA concentration would lead to an increase in the inference of spurious and contaminant sequences due to the lower signal-to-noise ratio but that the ASV methods would more accurately identify the true contamination present.

While other studies have investigated how sample biomass affects community composition estimates (22–24), to our knowledge, this is the first to have studied the impact of sample biomass on *in silico* community inference methods.

## RESULTS

### Experimental design.

Six 16S rRNA read clustering methods were chosen for comparison: two *de novo* OTU methods (UCLUST and UPARSE), three ASV methods (UNOISE, Deblur, and Divisive Amplicon Denoising Algorithm 2 [DADA2]), and an information-theoretic approach (Minimum Entropy Decomposition [MED]). Only methods that infer ASVs/OTUs *de novo* were selected, as *de novo* inference introduces less bias and generally accounts for more of the data. To the extent possible, each inference method was used in its default mode or with default parameters, along with its native chimera-removal function, as this represents the most likely usage by the typical user. Where no native chimera-removal tool existed, UCHIME (25) was used.

To assess the performance of the six selected methods, we first compared the methods on four high-biomass (undiluted) mock community data sets to show the baseline performance of each method on samples representative of high-microbial-biomass communities. Three of these data sets, referred to here as “Kozich,” “Schirmer,” and “D’Amore,” were from previously published studies (26–28), and the fourth data set, which we call “Zymo,” was generated for this study (see Table 1).

**TABLE 1:** High-microbial-biomass mock communities

Data set name (reference)	No. of strains	Genomic distribution	No. of raw reads
Kozich (26)	21	Uniform	269.8K
Schirmer (27)	57	Uniform	593.9K
D’Amore (28)	53	Log-normal	262.1K
Zymo	8	Uniform	427.2K

We next evaluated each method’s performance with varying microbial biomass by benchmarking each on a mock community dilution series. The dilution series mimics samples of successively lower biomass and allowed us to observe how each method’s inference results changed as biomass decreased.

### Evaluation.

To evaluate the results from each processing method, we classified ASVs/OTUs into five categories, using a scheme similar to that used previously by Edgar (29), Callahan et al. (14), and Nearing et al. (18). ASVs/OTUs that exactly matched a reference sequence from the known community were classified as “Reference” ASVs/OTUs. Those that differed from a more abundant Reference ASV/OTU by up to 10 nucleotides (nt) were labeled “Ref Noisy” ASVs/OTUs, as these likely represented reference-derived ASVs/OTUs incorrectly inferred as distinct due to sequencing errors (technical noise). The remaining ASVs/OTUs were compared to the National Center for Biotechnology Information’s Nucleotide (NT) database (30) using BLAST (31). Those that matched an NT sequence exactly were classified as “Contaminant” ASVs/OTUs, as these likely represented correctly identified contaminating DNA in the sample. ASVs/OTUs that differed from a Contaminant ASV/OTU by up to 10 nucleotides were dubbed “Contam Noisy”. All remaining ASVs/OTUs were labeled “Other” and might include unaccounted-for PCR artifacts (such as chimeras) and sequencing noise.

We further summarized results by computing recall and precision for the inferred ASVs/OTUs. Recall data measure the proportion of known community members detected by each method, while precision data give the proportion of predicted community members that belong to the known community. Precision was computed two different ways: first, by considering all reported ASVs/OTUs, where all non-Reference results represent false positives (FP); second, by considering only Reference and Ref Noisy results to represent true and FP, respectively (technical precision), as there is more ambiguity in the remaining categories and Contaminant ASVs/OTUs represent true positives in some contexts. These statistics give a sense of the accuracy of community diversity estimates. In addition, we computed the proportion of reads mapped to Reference ASVs/OTUs, which measures the overall effect of spurious ASV/OTU detection by an inference algorithm. Finally, we computed observed alpha diversities using three different indices and compared each to expected alpha diversities.

### High-microbial-biomass mock communities. (i) Total inferred ASVs/OTUs.

**FIG 1:**
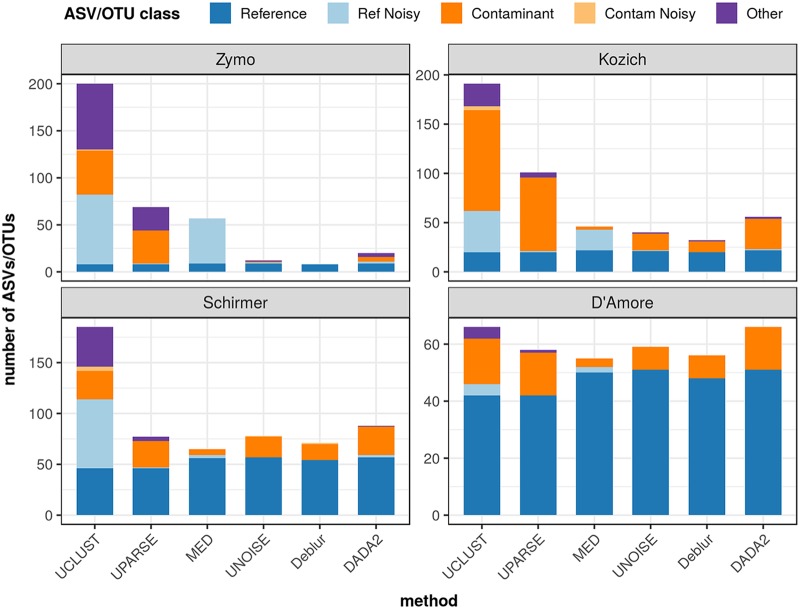
ASV/OTU classification of high-biomass samples. The composition of each high-biomass data set is indicated in terms of the number of ASVs/OTUs in each category, as inferred by each method (*x* axis). The categories are indicated as follows: Reference, exact match to a reference sequence from the known community; Ref Noisy, up to 10 nucleotides different from a reference sequence; Contaminant, exact match to an NT sequence; Contam Noisy, up to 10 nucleotides different from a contaminant; Other, any ASV/OTU not falling into these defined categories. Each panel shows sample compositions for one of the four high-biomass data sets. The *y* axis of each data set panel is scaled independently of those of the others.

With the four undiluted, high-biomass mock communities, the total number of distinct ASVs/OTUs inferred by each method varied widely (see Fig. 1). UCLUST reported the largest number of ASVs/OTUs on all data sets, while Deblur reported the fewest (for Zymo and Kozich) or second fewest (for Schirmer and D’Amore). MED found the fewest ASVs/OTUs on the Schirmer and D’Amore data sets but fell in the middle on the Zymo and Kozich data sets. Among the ASV methods, DADA2 detected the most ASVs.

### (ii) Classification of ASVs/OTUs.

The inference methods differed in their ability to detect the expected reference strains (Table 2; see also Table 3). All methods recovered nearly all references for the less diverse Zymo and Kozich data sets (8 of 8 for Zymo and at least 20 of 21 for Kozich, representing 100% and 95% recall, respectively), but for the larger Schirmer and D’Amore data sets, the OTU methods detected notably fewer references (46 of 57 for Schirmer and 42 of 53 for D’Amore, representing 81% and 79% recall, respectively). DADA2 and UNOISE detected the greatest number of reference strains in all data sets (96% to 100% recall), closely followed by MED (94% to 100% recall).

**TABLE 2:** Number of ASVs/OTUs in each category for the high-microbial-biomass mock communities

Data set	Method	No. of ASVs/OTUs^*b*^					
		Inferred total	Reference	Ref Noisy	Contaminant	Contam Noisy	Other
Zymo (8 strains)	UCLUST	200	8	74	47	1	70
	UPARSE	69	8	1	35	0	25
	MED	57	9^*a*^	48	0	0	0
	UNOISE	12	9^*a*^	1	1	0	1
	Deblur	8	8	0	0	0	0
	DADA2	20	9^*a*^	2	5	0	4

Kozich (21 strains)	UCLUST	191	20	42	102	4	23
	UPARSE	101	20	1	75	0	5
	MED	46	22^*a*^	21	3	0	0
	UNOISE	40	21	1	17	0	1
	Deblur	32	20	0	11	0	1
	DADA2	56	22^*a*^	1	31	0	2

Schirmer (57 strains)	UCLUST	185	46	68	28	4	39
	UPARSE	77	46	1	26	0	4
	MED	65	56	3	6	0	0
	UNOISE	78	57	0	20	1	0
	Deblur	71	54	0	16	1	0
	DADA2	88	57	2	28	0	1

D’Amore (53 strains)	UCLUST	66	42	4	16	0	4
	UPARSE	58	42	0	15	0	1
	MED	55	50	2	3	0	0
	UNOISE	59	51	0	8	0	0
	Deblur	56	48	0	8	0	0
	DADA2	66	51	0	15	0	0

a As some strains have more than one allele, the number of references detected may be greater than the total number of strains.

b Ref, Reference; Contam, Contaminant.

**TABLE 3:** ASV/OTU recall and precision for the high-microbial-biomass mock communities^*a*^

Method	Data set											
	Zymo			Kozich			Schirmer			D’Amore		
	Recall	Overall precision (%)	Technical precision	Recall	Overall precision (%)	Technical precision	Recall	Overall precision (%)	Technical precision	Recall	Overall precision (%)	Technical precision
UCLUST	100	4	10	95	10	32	81	25	40	79	64	91
UPARSE	100	12	89	95	20	95	81	60	98	79	72	100
MED	100	16	16	100	48	51	98	86	95	94	91	96
UNOISE	100	75	90	100	53	95	100	73	100	96	86	100
Deblur	100	100	100	95	63	100	95	76	100	91	86	100
DADA2	100	45	82	100	39	96	100	65	96	96	77	100

a Precision was calculated two ways. The first value counts all unexpected (non-Reference) ASVs/OTUs as false positives, whereas the second value counts only technical noise (Ref Noisy) as false positives.

Non-Reference ASVs/OTUs included the Ref Noisy, Contaminant, Contam Noisy, and Other categories described above. There was wide variation in the Ref Noisy category: UCLUST reported high numbers (42 to 74) of Ref Noisy ASVs/OTUs for three of the four mock communities, as did MED (21 to 48) for two data sets, whereas all other methods inferred no more than 2 Ref Noisy results. In general, several Contaminant ASVs/OTUs were detected. UCLUST and UPARSE gave the highest number (15 to 102) of Contaminant ASVs/OTUs, while MED identified the fewest (0 to 6). Among the ASV methods, DADA2 reported the most (5 to 31) and Deblur the fewest (0 to 16) Contaminant results. However, no method identified more than 4 Contam Noisy ASVs/OTUs. The number of inferred Other ASVs/OTUs typically ranged from 0 to 5, but UCLUST found much higher totals (23 to 70) for three communities, as did UPARSE (25) for the Zymo data set.

Owing to the wide variation in numbers of unanticipated, non-Reference ASVs/OTUs, precision varied greatly across methods and data sets (Table 3). Deblur and UNOISE gave relatively high precision (63% to 100% and 53% to 86%, respectively) on all data sets, as did MED on the Schirmer and D’Amore communities (86% and 91%), whereas UCLUST and UPARSE ranked last on all data sets (4% to 64% and 12% to 72%, respectively). MED exhibited the most variation across data sets, ranging from 16% to 91% precision. Technical precision (which counts only Ref Noisy results as false positives) was necessarily higher, with a large increase for UPARSE, but otherwise, the same general trends were observed among the methods.

### (iii) ASV/OTU abundance.

**FIG 2:**
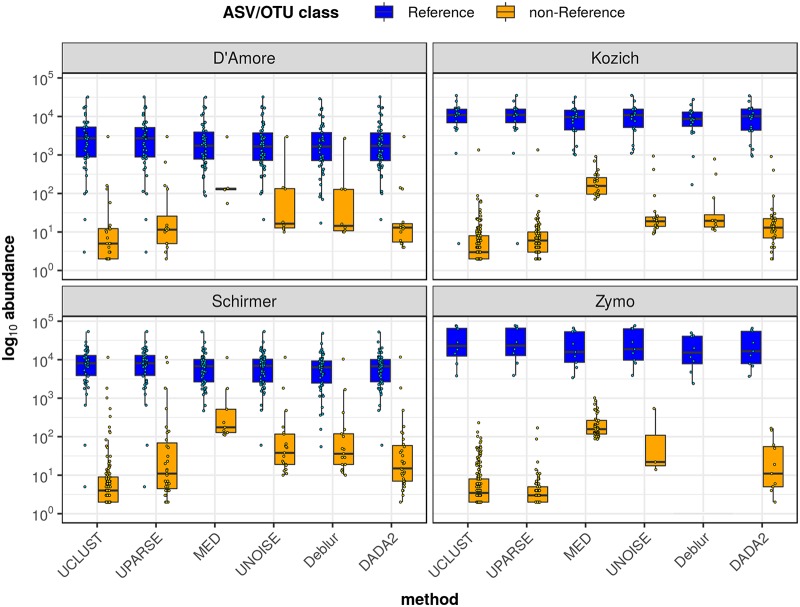
Abundance distributions of Reference and non-Reference ASVs/OTUs for high-biomass communities. Data represent log_10_-transformed abundance distributions of Reference ASVs/OTUs (those that match the 16S rRNA sequence of a known mock community member) and non-Reference ASVs/OTUs, as inferred by each of the six methods. Box plots show median, interquartile range (IQR), and 1.5 × IQR data. Individual ASV/OTU data points are overlaid on the box plots. Each subplot shows abundance distributions for one of the four high-biomass communities.

To measure the overall impact of the various noise sources on inference with respect to the target community, we computed the percentage of output reads assigned to Reference ASVs/OTUs for each method (see Table 4). For all high-biomass data sets, a large majority of reads (95.6% to 100%) were mapped to the target mock community regardless of the inference method. The proportion of reads assigned to each ASV/OTU category is shown in Fig. S1 in the supplemental material. We also plotted abundance distributions of Reference and non-Reference ASVs/OTUs, presented in Fig. 2, which shows how well the target community and unexpected ASVs/OTUs are separated in terms of signal strength.

**TABLE 4:** Percentage of sequence reads mapped to Reference ASVs/OTUs in high-biomass samples

Method	% sequence reads mapped to Reference ASVs/OTUs			
	Zymo	Kozich	Schirmer	D’Amore
UCLUST	99.2	98.9	96.6	98.3
UPARSE	99.8	99.1	95.2	98.0
MED	95.6	97.6	96.9	98.3
UNOISE	99.8	99.3	97.0	98.3
Deblur	100.0	99.3	96.9	98.3
DADA2	99.8	99.2	96.9	98.3

10.1128/mSystems.00163-18.4 FIG S1 Composition of high-biomass samples. Compositions are represented in terms of the relative abundances of reads assigned to ASVs/OTUs in each category for the four high-biomass mock communities. Categories are defined in Materials and Methods. Each panel shows sample compositions for one of the four high-biomass data sets. Download FIG S1, TIF file, 0.6 MB. Copyright © 2019 Caruso et al. 2019 Caruso et al. This content is distributed under the terms of the Creative Commons Attribution 4.0 International license.

### (iv) Alpha diversity.

**FIG 3:**
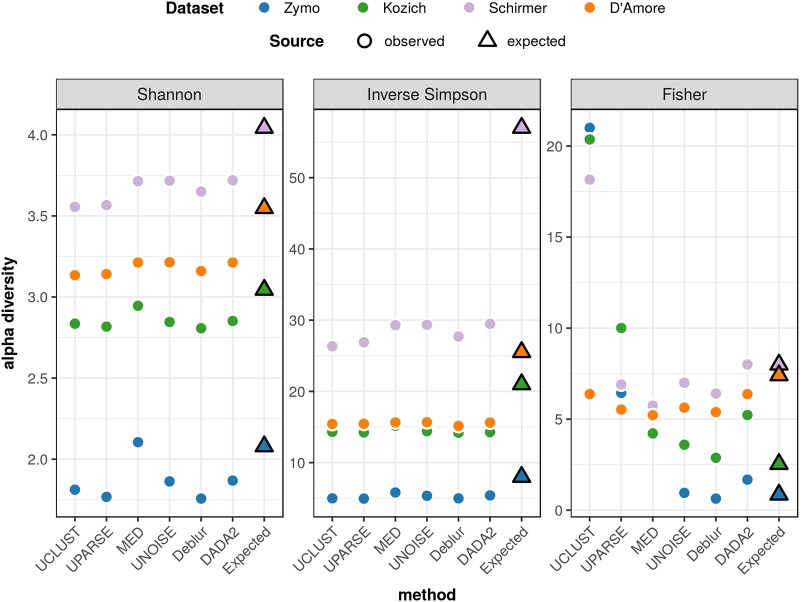
Alpha diversity of high-biomass samples. Shannon, inverse Simpson, and Fisher alpha diversity indices for each of the four high-biomass communities, computed from the ASVs/OTUs inferred by each of the six inference methods, are shown. Observed (inferred) alpha diversities are shown as round dots, and expected alpha diversities, determined on the basis of the genomic proportions of each strain used to construct the communities, are shown as triangles. Each alpha diversity measure has a distinct scale.

Shannon, inverse Simpson, and Fisher indices for alpha diversity, computed for each method’s inferred ASVs/OTUs, are plotted in Fig. 3. With the Shannon and inverse Simpson indices, all methods gave the diversity ranking that we would expect, given each community’s known richness and evenness (see Table 1), with somewhat higher diversities for the more sensitive MED, UNOISE, and DADA2 methods. With the Fisher index, only the ASV methods gave the expected ranking, while the other three methods gave inflated values for one or more data sets.

### Dilution series of Zymo mock community.

A summary of ASVs/OTUs inferred by each method for a subset of dilution series samples, including classification results, is shown in Table 5. Full results for all samples are given in Table S1 in the supplemental material.

**TABLE 5:** Number of ASVs/OTUs in each category for selected samples of the dilution series mock community benchmark

Dilution	Method	No. of ASVs/OTUs					
		Inferred-total	Reference	Ref Noisy	Contaminant	Contam Noisy	Other
1:1 (neat) (243.5K reads)	UCLUST	202	8	74	47	0	73
	UPARSE	69	8	1	35	0	25
	MED	57	9	48	0	0	0
	UNOISE	12	9	1	1	1	0
	Deblur	8	8	0	0	0	0
	DADA2	20	9	2	5	1	3

1:9 (282.0K reads)	UCLUST	450	8	62	218	25	137
	UPARSE	288	8	0	197	2	81
	MED	78	9	63	6	0	0
	UNOISE	119	9	0	97	3	10
	Deblur	85	8	0	75	0	2
	DADA2	114	9	2	91	0	12

1:81 (243.5k reads)	UCLUST	336	8	23	200	14	92
	UPARSE	269	8	1	186	2	72
	MED	153	9	65	76	2	1
	UNOISE	449	9	1	277	91	71
	Deblur	339	8	0	237	38	56
	DADA2	261	9	1	195	9	48

1:729 (144.3K reads)	UCLUST	377	8	2	239	37	91
	UPARSE	304	8	0	228	5	63
	MED	570	9	29	349	139	44
	UNOISE	530	9	0	330	123	68
	Deblur	430	8	0	293	68	61
	DADA2	381	9	1	270	49	52
							
1:6561 (49.4K reads)	UCLUST	195	8	2	127	9	49
	UPARSE	183	8	1	126	2	46
	MED	325	9	24	177	64	51
	UNOISE	267	9	3	161	39	55
	Deblur	226	9	1	152	16	48
	DADA2	193	8	1	129	11	44

10.1128/mSystems.00163-18.7 TABLE S1 ASVs/OTUs in each class for each method over all nine Zymo mock community dilution series samples. Download Table S1, CSV file, 0.00 MB. Copyright © 2019 Caruso et al. 2019 Caruso et al. This content is distributed under the terms of the Creative Commons Attribution 4.0 International license.

### (i) Total inferred ASVs/OTUs.

**FIG 4:**
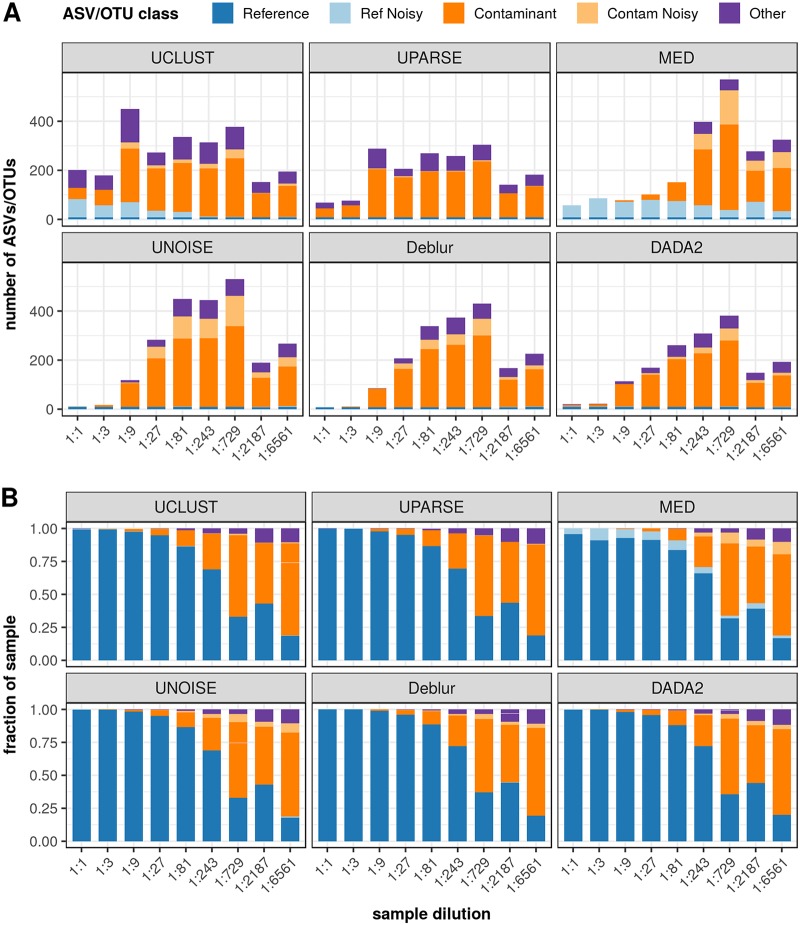
ASV/OTU classification of dilution series samples. (A) Classification of dilution sample in terms of the number of ASVs/OTUs in each category, as sample concentration decreases (*x* axis). (B) The composition of each dilution sample in terms of the relative abundance of sequences in each category. The categories are indicated as follows: Reference, exact match to a reference sequence from the known community; Ref Noisy, up to 10 nucleotides different from a reference sequence; Contaminant results, exact match to an NT sequence; Contam Noisy, up to 10 nucleotides different from a contaminant; Other, any ASV/OTU not falling into these defined categories. Each panel shows sample compositions inferred by one of the six inference methods.

As starting microbial biomass decreased, the total number of inferred ASVs/OTUs increased for all methods, dramatically for some (see Fig. 4A). This trend appeared not to hold for the two most dilute samples, but the deviation can be explained by the much lower sequencing depth obtained for these two samples—less than 50K reads each, compared to greater than 140K reads for each of the other samples. When inferred ASV/OTU totals were normalized by sample read count, the trend of increasing numbers of ASVs/OTUs was observed across the full dilution series (see Fig. S2).

10.1128/mSystems.00163-18.5 FIG S2 Total ASVs/OTUs inferred by each method at each concentration, normalized by median sample read count. For each dilution sample, the total number of inferred ASVs/OTUs was multiplied by the total number of output reads for a given method, and the result was then divided by the median number of output reads among all samples for that method. Each color corresponds to a different method. Download FIG S2, TIF file, 0.7 MB. Copyright © 2019 Caruso et al. 2019 Caruso et al. This content is distributed under the terms of the Creative Commons Attribution 4.0 International license.

At the highest concentrations (1:1 and 1:3), the ASV methods reported the fewest ASVs/OTUs (8 to 22), with the number of ASVs detected increasing steadily across the dilution series to a peak of 381 to 530 at a 1:729 dilution (the two most dilute samples were an exception, as explained above). With MED, the total numbers reported at higher concentrations were greater than those seen with the ASV methods but lower than those seen with the OTU methods for the undiluted sample, remaining relatively steady over the first four dilution samples (57 to 102 ASVs/OTUs); however, the MED total rose sharply such that the method detected the highest totals (278 to 570) for the three most dilute samples. In contrast, the totals reported by the OTU methods were at the high end for the three highest-concentration samples (69 to 288 for UPARSE and 202 to 450 for UCLUST), with a sharp spike for the 1:9 sample, but their totals leveled off over the rest of the dilution series, with UPARSE reporting the fewest ASVs/OTUs (142 to 304) for the four most dilute samples.

### (ii) Classification of ASVs/OTUs.

**FIG 5:**
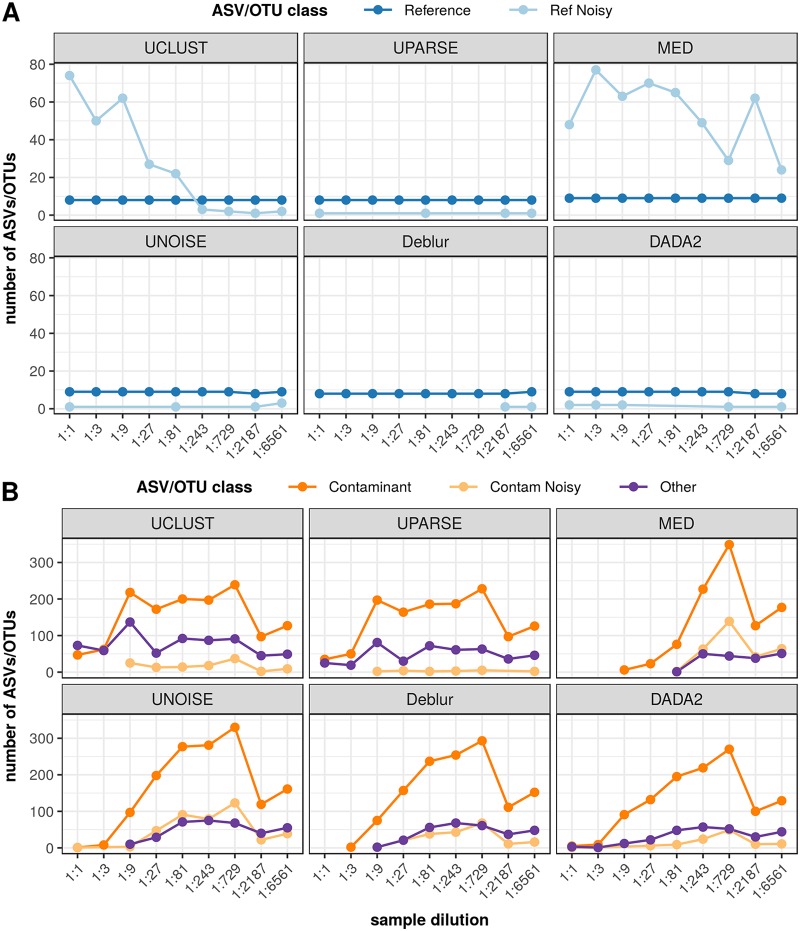
Trend lines of inferred ASVs/OTUs across the dilution series. (A) Number of Reference and Ref Noisy ASVs/OTUs inferred versus decreasing sample concentration. (B) Number of Contaminant, Contam Noisy, and Other ASVs/OTUs inferred versus decreasing sample concentration. ASV/OTU categories are defined in Materials and Methods. Each panel shows trend lines for ASVs/OTUs inferred by one of the six inference methods.

All methods detected all 8 expected community members, regardless of the sample dilution. Similarly to the high-microbial-biomass community results, MED and UCLUST inferred a high number of Ref Noisy ASVs/OTUs, but whereas the number remained high for MED across the dilution series, it declined for UCLUST at the lowest concentrations (see Fig. 5A).

The variation in total ASVs/OTUs was largely driven by Contaminant ASVs/OTUs (see Fig. 4), which increased in number as the samples became more dilute. However, the trend lines indicated in Fig. 5 show that while this increase was approximately linear for the ASV methods, it was less linear for MED and was not linear at all for the OTU methods, which exhibited the least association between sample dilution and the number of Contaminant results. We observed a smaller but similar trend for Contam Noisy ASVs/OTUs with the ASV methods and MED. The remaining Other ASVs/OTUs showed less association with dilution for the ASV methods and no association for the OTU methods and MED. We also found that among the ASV methods, DADA2 typically reported the fewest Contaminant, Contam Noisy, and Other ASVs/OTUs.

Recall was perfect across the dilution series; all methods detected all reference strains at every sample concentration. However, precision was more variable, reflecting the high numbers of non-Reference ASVs/OTUs seen at lower concentrations. For the two highest concentrations, the ASV methods exhibited much higher precision (36% to 100%) than other methods; for the remaining dilution samples, all methods exhibited precision levels below 10%.

### (iii) ASV/OTU abundance.

The proportion of reads in each category for each method across the dilution series is shown in Fig. 4B, illustrating the impact of contamination as biomass decreased. We show that as concentration dropped, the proportion of Reference reads declined considerably with all methods to less than 20% for the most dilute sample. At the highest concentrations (1:1 and 1:3), Deblur and the OTU methods assigned over 99% of reads to Reference ASVs/OTUs. MED was the only method to assign a notable proportion (2% to 11%) to Ref Noisy across all dilutions. Beginning with the 1:9 dilution sample, reads assigned to Contaminant ASVs/OTUs became apparent with all methods, increasing steadily in proportion until they dominated the inferred composition for the three lowest dilutions, where they made up 43% to 70% of the sample. Reads from Other and Contam Noisy ASVs/OTUs also generally increased across the dilution series. The former typically comprised a larger fraction, reaching a maximum of ∼10% at the lowest concentration.

**FIG 6:**
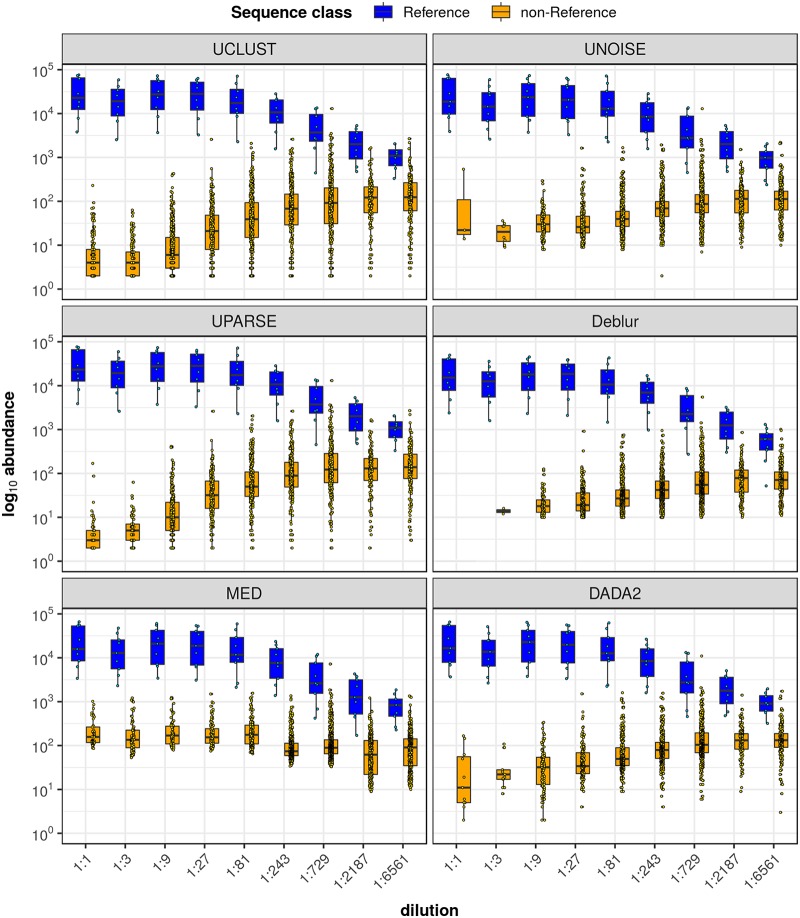
Abundance distributions of Reference and non-Reference ASVs/OTUs for the dilution series. Data represent log_10_-transformed abundance distributions of Reference ASVs/OTUs (those that match the 16S rRNA sequence of a known mock community member) and non-Reference ASVs/OTUs versus decreasing community biomass for the Zymo mock community. Box plots show median, IQR, and 1.5 × IQR data. Individual ASV/OTU data points are overlaid on the box plots. Each subplot shows abundance distributions inferred by one of the six methods benchmarked.

As with the undiluted mock communities, we compared the distribution of the Reference abundances (i.e., target community, or signal) to that of the non-Reference abundances (noise) for the dilution series samples. Results are shown in Fig. 6. Regardless of inference method, we observed that as the concentration decreased, the Reference and non-Reference distributions steadily converged—signal weakened while noise grew. Over the first several dilutions (1:1 to 1:81 relative concentrations), the signal distribution remained nearly constant, and even though the strength of the noise increased, signal and noise data were generally well separated. However, with decreasing DNA concentrations, the distributions overlapped considerably, as the signal strength steadily declined while the noise strength tended to increase. At very low concentrations, there was little distinction between the overlap observed with the various methods.

### (iv) Alpha diversity.

We again computed alpha diversities for each dilution sample (plotted in Fig. S3). With the Shannon and inverse Simpson indices, diversity estimates increased as sample biomass decreased (with the exception of the second-to-last dilution). The Fisher index more closely reflected the observed ASVs/OTUs: the ASV methods exhibited roughly linear increases in diversity, whereas the OTU methods showed no clear trend.

10.1128/mSystems.00163-18.6 FIG S3 Inferred alpha diversities for each dilution sample. Data points represent Shannon, inverse Simpson, and Fisher alpha diversity indices for each of the dilutions, computed from the ASVs/OTUs inferred by each of the six inference methods. As expected, alpha diversity values were inflated drastically with increasing dilution, likely due to the increased number of contaminants in the sample. Download FIG S3, TIF file, 0.2 MB. Copyright © 2019 Caruso et al. 2019 Caruso et al. This content is distributed under the terms of the Creative Commons Attribution 4.0 International license.

### (v) Pooled sample processing.

The results presented above were obtained by processing each dilution sample separately with each method’s standard workflow. However, we also varied the experiment by pooling the samples and then processing them together with each workflow, a strategy often used by researchers to process up to an entire sequencing run. In general, the total number of inferred ASVs/OTUs for each sample, as well as the counts in each category, increased when ASVs/OTUs were inferred from pooled samples. An exception to this trend was seen with DADA2, because its workflow processes samples individually by default. This indicates that results from most methods are data set dependent; i.e., inference results for a sample vary depending on which other samples are processed with it.

## DISCUSSION

### High-microbial-biomass benchmarking.

With the high-biomass mock communities, the three ASV methods agreed closely on the numbers of ASVs inferred in each category. More importantly, the compositions corresponding to each sample as inferred by the ASV methods were closer to the known diversity of each community than were the compositions inferred by OTU methods. This observation holds in terms of estimated richness (i.e., numbers of ASVs/OTUs inferred) as well as in terms of alpha diversity (Fig. 3), which takes into account ASV/OTU abundances. The ASV methods also outperformed other methods in terms of their ability to distinguish the true signal from noise.

Deblur exhibited the best specificity (fewest non-Reference ASVs/OTUs), but DADA2 and UNOISE had better sensitivity for Reference ASVs/OTUs. These results are consistent with the findings of Nearing et al. (18), who reported that DADA2 was the most sensitive and Deblur the most specific among the ASV methods. The higher number of Contaminant results detected by DADA2 and UNOISE suggest that they may also be more sensitive to low-abundance contamination, but it is possible that they underestimated sequencer error, such that some community-derived sequences with several errors fell outside the error model as well as the definition of Ref Noisy and were reported as Contaminant results. Thus, the choice of which ASV method to use depends upon the goals of the research. If minimizing detection of spurious ASVs/OTUs is most important, Deblur may be the more appropriate choice. However, if maximizing detection of true community members and/or of biological contaminants is a priority, DADA2 or UNOISE appears to be the better choice.

Results for MED were less consistent. While sensitivity was high for all high-biomass samples, specificity was poor for the Zymo and Kozich communities due to a large number of Ref Noisy ASVs/OTUs, resulting in inflated alpha diversity estimates, especially for the Zymo community. Most of these Ref Noisy results were within 1 nt of a Reference result, evidence that they represented false positives that should have clustered with a Reference ASV/OTU. This is also reflected in the signal and noise distributions: MED’s inference of several Ref Noisy instances with relatively high abundance resulted in the poor separation of signal from noise observed with the Zymo and Kozich data sets (Fig. 2). Hence, although MED was designed to distinguish biological strains with extremely similar 16S sequences, it is prone to reporting spurious ASVs/OTUs that arise from sequencer errors.

The OTU methods gave both the poorest recall and the poorest specificity, which supports the findings of Callahan et al. (14) and Nearing et al. (18). This relative inaccuracy produced both some of the lowest Shannon and inverse Simpson diversity estimates along with greatly inflated Fisher estimates. Since OTU methods rely only on a distance metric for clustering, their reduced sensitivity results from lumping together distinct strains with high 16S sequence similarity, ignoring sequence abundance. On the other hand, the high numbers of non-Reference OTUs reported by the OTU methods likely represent the result of splitting sequences into distinct clusters due to sequencing errors that fall outside the similarity threshold of the true template. UPARSE was identical to UCLUST in sensitivity but showed better specificity in terms of Ref Noisy OTUs. This is best explained by UPARSE’s strict quality filtering step prior to cluster inference, which removes the majority of reads that contain several sequencing errors.

Both OTU methods were notable for reporting high numbers of Contaminant OTUs and Other OTUs, some of which likely reflect real contamination. However, UCLUST and UPARSE diverged most from the consensus on the Zymo and Kozich data sets, which had poorer read quality profiles than the Schirmer and D’Amore data sets (data not shown) and thus had more sequencing errors. Clearly, the fixed similarity threshold of OTU methods is ill-suited to dealing with such scenarios. In addition, UCLUST and UPARSE reported one or more Reference OTUs with much lower signal strength than that reported by the other methods (see Fig. 2). We can conclude that OTU methods are inferior to the other algorithms on multiple counts.

### Dilution series benchmarking.

The dilution series results clearly show that as the starting DNA concentration decreased, the ASVs/OTUs derived from noise sources comprised an increasing proportion of the inferred community, in terms of both the number of distinct ASVs/OTUs and their abundances. The dramatic rise in the numbers of Contaminant ASVs/OTUs detected across the methods at lower starting concentrations suggests that there are many more contaminating species present at detectable levels in sequencing libraries prepared from samples with low microbial biomass. This makes sense: when low levels of sample DNA are present for PCR amplification, lower levels of microbial contaminants (e.g., those from nonsterile laboratory equipment and reagent kits [21]) make up a larger proportion of the total DNA. Hence, contaminant sequences are amplified to a greater extent than in high-microbial-biomass samples, where the sample DNA overwhelms the contamination. Contaminant-labeled ASVs/OTUs detected in the low-microbial-biomass dilution samples thus likely reflect biological contamination introduced during sample processing. As a consequence, alpha diversity estimates (see Fig. S3 in the supplemental material) depend to some extent on sample biomass, and this effect may be quite pronounced for very-low-biomass samples.

### (i) Algorithm performance.

The strong association between sample dilution and the number of Contaminant results inferred by the ASV methods is evidence that these methods detect true contamination more accurately than the OTU methods or MED. This is also reflected in the higher sensitivity and precision achieved by these methods for the high-microbial-biomass communities. The differences between the ASV methods in numbers of Contaminant and Contam Noisy ASVs/OTUs, which maintain their order across most of the dilution series (i.e., UNOISE reports more than Deblur, which reports more than DADA2), can be attributed to differences in sequence-error models. Either UNOISE and Deblur are more sensitive than DADA2 for these samples or UNOISE and Deblur underestimate the actual sequencing error, leading to poorer precision. The latter seems more likely, since DADA2 estimates its error model dynamically from the data whereas UNOISE and Deblur use a fixed error model. Since the dilution series data set had relatively poor read quality, it is likely that DADA2’s model is better adjusted to the higher degree of error and that UNOISE and Deblur underestimate the error profile on this data set. Changing the default error model parameters for UNOISE and Deblur might yield better results for data sets with lower read quality, but establishment of guidelines for doing so may be challenging and is beyond the scope of this study.

MED, the entropy-based inference method, is unique in inferring no or very few Contaminant results at higher concentrations, with the number of Contaminant results rising sharply such that MED infers the greatest proportion at very low concentrations. This phenomenon can be explained by MED’s use of a sequence abundance threshold (0.02% of total data set reads by default) to filter out clusters arising from noise. The filter greatly limits the number of Contaminant ASVs/OTUs reported at higher concentrations, when contamination is least amplified, but as the proportion of contaminant DNA rises, many more contaminant sequence abundances exceed the abundance filter value. Thus, the effectiveness of this type of filter for removing biological noise depends unpredictably on the sample’s microbial DNA concentration (and on the data set size), and this approach also risks removing low-abundance species present in the target community, reducing sensitivity. The relatively high numbers of Ref Noisy results inferred by MED at all sample dilutions, and the high numbers of Contaminant results and Contam Noisy results inferred at low concentrations, show that the entropy criterion used to divide sequence clusters is too sensitive; it underestimates sequencer error, resulting in many false positives. MED’s default entropy criterion could be adjusted to better reflect the error rate for a given data set, but choosing an appropriate value would require validation by the user.

To some extent, the inference results for the OTU methods followed the trend of increasing numbers of OTUs with decreasing concentrations, driven by a rise in Contaminant OTUs. However, the number of inferred Contaminant results was associated with sample biomass to a much lower degree than was observed for the ASV methods. As sample concentration decreases and more contaminating sequences are amplified, the similarity threshold used with the OTU methods may lump some distinct contaminant sequences together, leading to the observed plateau in Contaminant sequences. The same mechanism explains the reduced sensitivity observed previously with the high-microbial-biomass communities.

With UCLUST, the drop in the number of Ref Noisy results over the dilution series reflects the OTU clustering strategy. For the highest-concentration samples, large quantities of community template DNA led to high sequencing depth and a corresponding long tail in the sequence error distribution for these sequences, producing many Ref Noisy OTUs due to reads with errors that fall outside the OTU similarity threshold. Decreased concentrations reduce the sequencing depth and hence lower the absolute number of errors per sequence; thus, fewer community-derived sequences have errors that escape the similarity threshold. This phenomenon clearly illustrates why OTU methods in general are not well suited to use with high-throughput sequencing data: the typical similarity threshold value does not account for the wide error distributions that occur with deep 16S rRNA sequencing; however, increasing the threshold would only further degrade the already reduced sensitivity.

### (ii) Identification of contaminants.

The significant overlap of the signal and noise abundance distributions (see Fig. 6) at low DNA concentrations illustrates the difficulty of separating the target community from noise sources for low-microbial-biomass samples. An abundance filter (such as that employed by MED) is ineffective in this scenario, as any choice of threshold risks either removing community species or retaining noncommunity species. Since this overlap was observed for all inference methods at low concentrations, none of these methods is sufficient on its own to adequately distinguish community signal from noise.

However, the clear positive correlation of the starting DNA concentration with signal strength and the negative correlation with noise strength suggest a possible strategy for identifying contaminant sequences: namely, a dilution series could be prepared from a mock community or sample aliquot and sequenced as a positive control with the samples under study. Inferred ASVs/OTUs whose abundance increased with decreasing concentration in the control samples could then be labeled as contaminants and removed from the inferred communities of the study samples. Indeed, such an approach has already been implemented in one form by Davis et al. (32), and other variations on this approach are possible. Furthermore, on the basis of the results of this study, such a strategy would work best when an ASV method is used for sequence inference, since the ASV algorithms produced the best correlation between the concentrations and both the number and abundance of noisy ASVs/OTUs (see Fig. 5 and 6). ASV methods were also best at limiting the number of Ref Noisy ASVs/OTUs, which is important since these would not be inferred as contaminants using the approach described above.

### (iii) Reproducibility of sample inference.

Although not strictly related to microbial biomass, the dependence of most methods’ inferences on whether samples were processed separately or pooled gives further insight into their performance. UCLUST, UPARSE, UNOISE, and MED all infer clusters based on the entire input data set, regardless of the number of samples; reads from each sample are then mapped to clusters to obtain sample-wise ASV/OTU abundances. However, employing this strategy may mean that community inference is difficult to reproduce, as it depends on the number of samples processed together. In contrast, Deblur and DADA2 perform inference on each sample independently by default.

After initial inference, UNOISE, MED, and Deblur all attempt to control false positives using a minimum-abundance filter for the full data set. UNOISE and Deblur employ absolute minima, whereas MED uses a relative minimum. In either case, these abundance filters complicate sample inference by creating a dependence on data set size and may remove rare species. In Deblur and UNOISE, the use of such a filter may also give an overly optimistic estimate of specificity—here, pooled sample processing showed that Deblur actually inferred several ASVs that were filtered out when samples were processed independently, due to the smaller data set size. The only inference method that is data set independent (i.e., for which sequence inference results for a sample do not vary depending on which other samples are processed with it) is DADA2, which represents a significant strength for reproducibility.

### (iv) Limitations.

A few factors in our study limit the conclusions we can draw. First, the Zymo community has low diversity and uniform genomic proportions and thus is not representative of a typical microbiome sample. This mock community clearly exhibits the impact of contaminant noise at low microbial biomass, but the high proportion of each reference species did not challenge the sensitivity of inference at lower DNA concentrations. Dilution series data from a more varied community structure might show further distinctions between inference methods, particularly among the ASV algorithms. Another limitation is that a dilution series cannot perfectly mimic low-microbial-biomass samples, as the act of dilution may itself introduce noise in the form of contamination. The dilution series does provide a good approximation of the outsized effect that even low levels of contamination can have when the starting DNA concentration is low. As a third caveat, there is inevitably some overlap between the ASV/OTU categories that represent technical noise (the Ref Noisy and Contam Noisy categories) and those representing other noise sources (the Contaminant and Other categories). For example, BLAST analyses against NT represent an imperfect way to identify contaminants; some hits may in fact be products of sequencer error corresponding to a more prominent strain, and not every potential contaminant is catalogued in NT. Nevertheless, this scheme provides a consistent, logical framework within which to compare and assess inference methods and demonstrates clear differences between the methods studied. Finally, published reference sets may contain errors or omissions. This could lead to underestimation of the recall and precision of Reference ASVs/OTUs and to greater overlap of signal and noise abundance distributions.

### Conclusions.

Despite these limitations, our observations indicate that contaminants can be a considerable confounding factor for low-microbial-biomass samples. In sequencing the 16S rRNA gene of low-biomass communities, we showed that sequences representing DNA contamination can be amplified such that their abundance becomes comparable to that of the target community sequences. We have also demonstrated that none of the inference methods studied here is sufficient on its own to distinguish the target community from biological contamination in this scenario. More research is needed to develop reliable techniques for removing contamination, and since perfectly aseptic sample processing is not realistic, contaminant removal by *in silico* methods may be more effective. We found that when challenged with technical noise, ASV methods did the best job of limiting or eliminating false positives due to sequencer error. In addition, the ASV methods exhibited sensitivity that was equal to or better than that of other methods, particularly the OTU methods. Thus, due to their superior sensitivity and specificity, we recommend the use of ASV methods for processing 16S rRNA sequence data. We further hypothesize that an ASV method combined with a dilution series as a positive control may provide a viable tool for detecting contaminants, and we propose this for future study.

## MATERIALS AND METHODS

### Data sets.

The Kozich mock community (26) comprises equal concentrations of 21 different bacterial strains. The Schirmer mock community (27) was made from equal proportions of 57 prokaryotic strains (both archaea and bacteria). The D’Amore mock community (28) was made from the same 57 strains as the Schirmer community, but the D’Amore sample has DNA quantities that vary according to a logarithmic distribution, and in the sample chosen for this study, only 53 of the 57 strains actually appeared in the raw sequence reads. The Zymo community includes 8 strains with equal genomic proportions. Table 1 gives a summary of the high-microbial-biomass mock communities. See “Data availability” below for how to access all data sets and reference sequences.

The Zymo mock community, including the high-biomass (undiluted) sample and each of the dilution series samples, comprises 8 bacterial strains in equal proportions (see Table 6 for community composition) and was prepared for this study from the ZymoBIOMICS Microbial Community Standard (lot number ZRC183430) (available from Zymo Research). This mock community consists of both Gram-positive and Gram-negative bacteria in addition to two yeast (Saccharomyces cerevisiae and Cryptococcus neoformans) species and thus is a useful tool to ensure the success of DNA extraction (and subsequent sequencing) from a broad diversity of microorganisms which may have intrinsic biological properties (e.g., cell wall thickness) that make them more or less refractory to DNA isolation. DNA was extracted from the microbial standard by the use of a Qiagen DNeasy blood & tissue kit following the manufacturer’s recommended protocol. In brief, bacterial cells were subjected to mechanical and enzymatic lysis, followed by removal of the inhibitor by precipitation. DNA was collected by passing it through a DNA binding column. For further purification, binding products were washed to remove contaminants, and purified DNA was collected by elution.

**TABLE 6:** Composition of the ZymoBIOMICS microbial community standard

Species	Gram stain result	% genomic DNA abundance
Pseudomonas aeruginosa	−	12
Escherichia coli	−	12
Salmonella enterica	−	12
Lactobacillus fermentum	+	12
Enterococcus faecalis	+	12
Listeria monocytogenes	+	12
Bacillus subtilis	+	12
Escherichia coli	+	12
Saccharomyces cerevisiae^*a*^	Yeast	2
Cryptococcus neoformans^*a*^	Yeast	2

a These species were not sequenced, as they lack the 16S rRNA gene.

Subsequently, eight serial dilutions were made from the extracted DNA, where each successive aliquot was diluted with molecular-grade water to 1/3 of its previous concentration, resulting in a total of nine samples with the following concentrations relative to the original extraction: 1, 1/3, 1/9, 1/27, 1/81, 1/243, 1/729, 1/2187, and 1/6561. The V4 region of the 16S rRNA gene was amplified by PCR using Golay barcodes and the 515FB-806RB primer pair (33–37). PCR was performed in triplicate for 35 cycles with ProMega hottaq polymerase (M5005), and amplification products were confirmed with gel electrophoresis. Amplified DNA was purified with a Qiagen QIAquick PCR purification kit. Samples were normalized to a concentration of 10 ng/µl, pooled, and sequenced on an Illumina MiSeq instrument using Reagent kit V2 to generate 2 × 251 base-pair reads.

### Sequence preprocessing.

Prior to clustering, reads were trimmed, merged, and filtered to remove low-quality data. In all data sets, the first 15 nucleotides from the 5′ end, which often contain pathological errors, were removed, as were the low-quality 3′ tails, which varied by data set (trim positions of forward/reverse reads for Zymo, 230/210; for D’Amore, 250/240; for Kozich, 240/220; for Schirmer, 240/220). After trimming, forward and reverse reads were merged and then filtered to remove low-quality sequences. Merging was performed with the USEARCH *fastq_mergepairs* command, with a maximum of 10 differences (*fastq_maxdiffs* = 10). To ensure that only sequences from the V4 region of the 16S small subunit (SSU) rRNA were retained, merged sequences were removed if their lengths were outside the expected range for the primer pair used. These ranges are 220 to 225 bp for the Zymo and Kozich reads and 258 to 263 bp for the D’Amore and Schirmer reads (the latter used a different primer pair that targets a longer V4 sequence). Merged sequences were further filtered to remove those with more than 2 expected errors, based on the posterior Q-scores computed by USEARCH. An exception to this protocol is represented by the DADA2 pipeline, in which forward and reverse reads are filtered independently and merged only after ASV/OTU inference. In order to retain a proportion of the data similar to that for the other methods, forward and reverse reads were filtered with a higher maximum number of expected errors for the DADA2 pipeline (forward/reverse maximum errors for Zymo, 2.5/2.5; for D’Amore, 2.5/2.5; for Kozich, 2.5/3.0; for Schirmer, 2.5/2.5).

### Sequence inference methods.

Between the two *de novo* OTU methods included, UCLUST (38) version 1.2.22 was chosen because it has been widely used in previous microbiome research and UPARSE (29) was selected because it may greatly reduce the inflation of community richness estimates that result from most OTU clustering algorithms (39). Three ASV methods, UNOISE (15), Deblur (16), and DADA2 (14), were included because they were the only published stand-alone ASV methods available at the time of writing. Finally, MED (40), which uses an information-theoretic approach, was included for its potential to give distinct results.

### (i) OTU methods.

The UCLUST algorithm of QIIME begins with an abundance-sorted list of sequences. It then uses a fast heuristic to align those sequences against a database of cluster seeds, which is initially empty. Sequences are greedily clustered if they are within the radius of variation (typically 3%, or 97% similarity) of existing seeds; otherwise, they become new seeds. UPARSE uses the same greedy-clustering strategy but precedes that with a stringent quality-filtering step and also removes chimeric sequences during the clustering stage when a query sequence is best explained as a chimera of existing seeds.

### (ii) ASV methods.

UNOISE assumes a parametric model for sequencing errors, where the maximum relative abundance β of a sequence with errors relative to its correct template sequence is given by the following function (15):

$$\beta (d)=\frac{1}{2^{\alpha d+1}}$$

where *d* is the Levenshtein distance from the true sequence and α is a tuning parameter (α = 2 by default). A sequence whose abundance value is consistent with (i.e., less than) another sequence’s error model is clustered with the more abundant sequence; otherwise, it is considered a distinct true sequence. Deblur instead uses a stepwise model in which the expected error frequencies at each Hamming distance from the template are specified individually. In decreasing order of abundance, each sequence’s expected error abundances are computed and subtracted from the abundances of neighboring sequences (up to a Hamming distance of 11). Sequences whose abundance remains above zero after all subtractions have been performed are inferred as the true ASVs. In contrast to the *a priori* error models of UNOISE and Deblur, DADA2 estimates its error model directly from the data using an iterative strategy. Beginning with a worst-case assumption for the error model, the algorithm alternates between clustering sequences given the error model and estimating the error model given the clustering until convergence occurs. During clustering, all sequences begin in a single cluster whose inferred template (centroid) sequence is the most abundant one, and the probabilities that all other sequences were derived from the true one, given the error model, is calculated. If the least-probable sequence is below a *P* value threshold (1 × 10^−40^ by default), it forms a new cluster centroid, and sequences are reassigned to their most likely cluster. This cluster division repeats until all clusters are consistent with the error model, and cluster centroids then become the inferred ASVs.

### (iii) Entropy method.

MED begins by placing all sequences in a single cluster and assumes that they are aligned by virtue of having a common primer (shorter sequences are simply padded with gaps). The Shannon entropy value is computed for each alignment column, and if any column has an entropy value higher than a threshold value (computed dynamically for each cluster), the cluster is divided so as to make the entropy value for the offending column zero in each new cluster. New entropy threshold values are computed, and cluster division repeats until all clusters have entropy values below the designated threshold. MED then removes those clusters whose abundance is below a minimum (0.02% of all data set reads by default), considering these to represent noise.

### (iv) Usage.

Each of the six clustering methods was run with default parameters on each of the preprocessed data sets. The primary commands used for each method, as well as any additional required parameters, are described here. For the UCLUST method, chimeras were first removed with *identify_chimeric_seqs.py* using the UCHIME method (*-m usearch61*) with the *gold.fa* reference database (available from http://drive5.com/otupipe/gold.tz). Sequences were then clustered *de novo* with the *pick_de_novo_otus.py* command and the default *uclust* algorithm. The UPARSE method was executed by calling the *cluster_otus* command in USEARCH (which concurrently removes chimeras) and then mapping the reads to cluster seeds with the *otutab* command. Similarly, the UNOISE method was run by calling *unoise3* in USEARCH (which also removes chimeras), and the reads were mapped to centroids with the *otutab* command. The MED method was run by invoking the *decompose* command within Oligotyping Pipeline software. As MED does not include native chimera removal, chimeras were removed with *uchime2_denovo* in USEARCH after clusters were sorted by size. Deblur was run by calling *workflow* within the Deblur package, with the *-t* (trim) option set to the lower bound of the merge length windows mentioned above to guarantee that all sequences would have the same length. The DADA2 method was run with a custom R script based on the dada2 library as follows. First, error rates were estimated with the *learnErrors* command; dereplicated reads were then clustered with *dada*, merged with *mergePairs*, and tabled with *makeSequenceTable*; sequences outside the allowed merge length window (see “Sequence preprocessing” above) were then removed, and chimeras were removed with *removeBimeraDenovo*.

### Analysis of inference results.

For recall and precision analyses, we used the following observation-versus-expectation criteria: ASVs/OTUs that were both expected and observed (i.e., observed Reference ASVs/OTUs) were true positives (TP), those expected but not observed (unobserved References) were false negatives (FN), and those observed but not expected (all non-References) were false positives (FP). In computing technical precision, only Ref Noisy results were counted as FP.

All analysis of clustering results was completed in R (41). The analysis code is available at https://github.com/lakarstens/noisy-microbes.

### Software used.

UCLUST was implemented with scripts from QIIME v1.9.1 (42). The *identify_chimeric_seqs.py* script requires USEARCH v6.1.544 (25), and *pick_de_novo_otus.py* calls the PyNAST alignment tool (v0.1) (43). UPARSE and UNOISE were implemented with USEARCH v10.0.240. For the MED pipeline, clustering was done with v2.1 of Oligotyping Pipeline software (40); however, chimera removal was done with USEARCH version 9.2.64 (due to a known bug in v10.0.240). The Deblur pipeline uses Deblur v1.0.3 (Amir et al. [16]), which depends on VSEARCH v2.5.0 (Rognes et al. [44]), MAFFT v7.3.10 (45), and SortMeRNA v2.0 (46). DADA2 was implemented in R with v1.6.0 of the dada2 package (14). All analysis of clustering results was completed in R v3.4.3 (41). The analysis and all pipeline scripts are available at https://github.com/lakarstens/noisy-microbes.

### Data availability.

The Kozich raw data set is available as run 130403 from the Mothur MiSeq development data website. Reference sequences are published on the same site. The Schirmer data set was obtained from the European Nucleotide Archive (ENA), project accession number PRJEB6244, run accession number ERR777695 (sample metaID-35). The D’Amore data are also available from ENA project accession number PRJEB6244, run accession number ERR777739 (sample metaID-88). Reference sequences for both the Schirmer and D’Amore data sets were obtained from the data repository for the DADA2 manuscript in the Stanford digital stacks, and their accuracy was confirmed through correspondence with R. D’Amore. Raw Illumina sequence reads from the Zymo mock community dilution series have been deposited in the Sequence Read Archive (SRA) under accession number SRP155048. Zymo reference sequences were obtained from Zymo Research. All reference sequence sets are also included in Text S1, Text S2, and Text S3 in the supplemental material.

10.1128/mSystems.00163-18.1 TEXT S1 fasta file containing the reference sequences for the Zymo mock community. Download Text S1, TXT file, 0.07 MB. Copyright © 2019 Caruso et al. 2019 Caruso et al. This content is distributed under the terms of the Creative Commons Attribution 4.0 International license.

10.1128/mSystems.00163-18.2 TEXT S2 fasta file containing the reference sequences for the Kozich mock community. Download Text S2, TXT file, 0.2 MB. Copyright © 2019 Caruso et al. 2019 Caruso et al. This content is distributed under the terms of the Creative Commons Attribution 4.0 International license.

10.1128/mSystems.00163-18.3 TEXT S3 fasta file containing the reference sequences for the Schirmer/D’Amore mock communities. Download Text S3, TXT file, 0.2 MB. Copyright © 2019 Caruso et al. 2019 Caruso et al. This content is distributed under the terms of the Creative Commons Attribution 4.0 International license.
