# Supplementary figures and images for: Performance of Microbiome Sequence Inference Methods in Environments with Varying Biomass
Source: mSystems. 2019 Feb 19;4(1):e00163-18. doi: 10.1128/mSystems.00163-18 (PMC6381225; doi:10.1128/mSystems.00163-18)

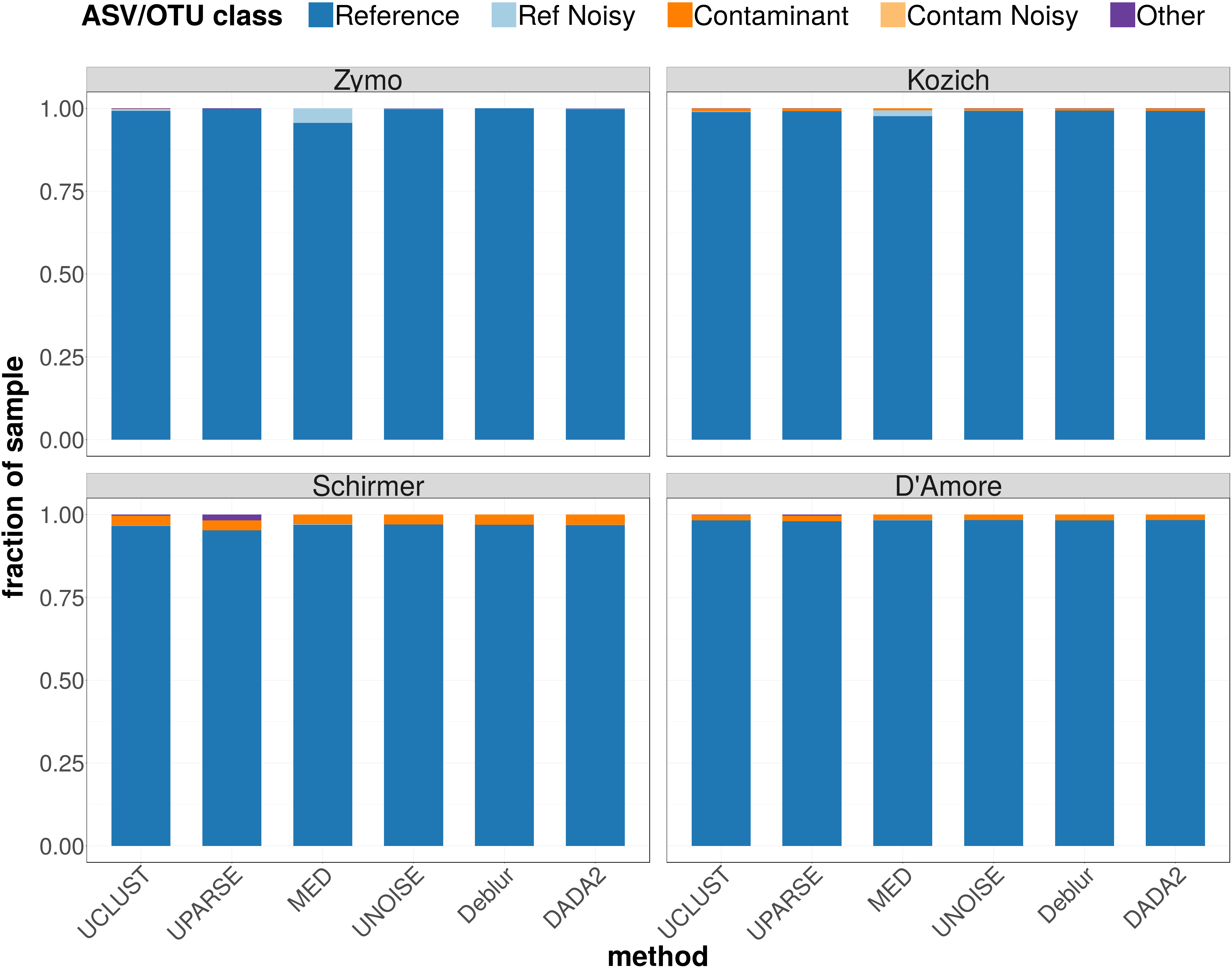

Supplement: FIG S1 [file mSystems.00163-18-sf001.tif]

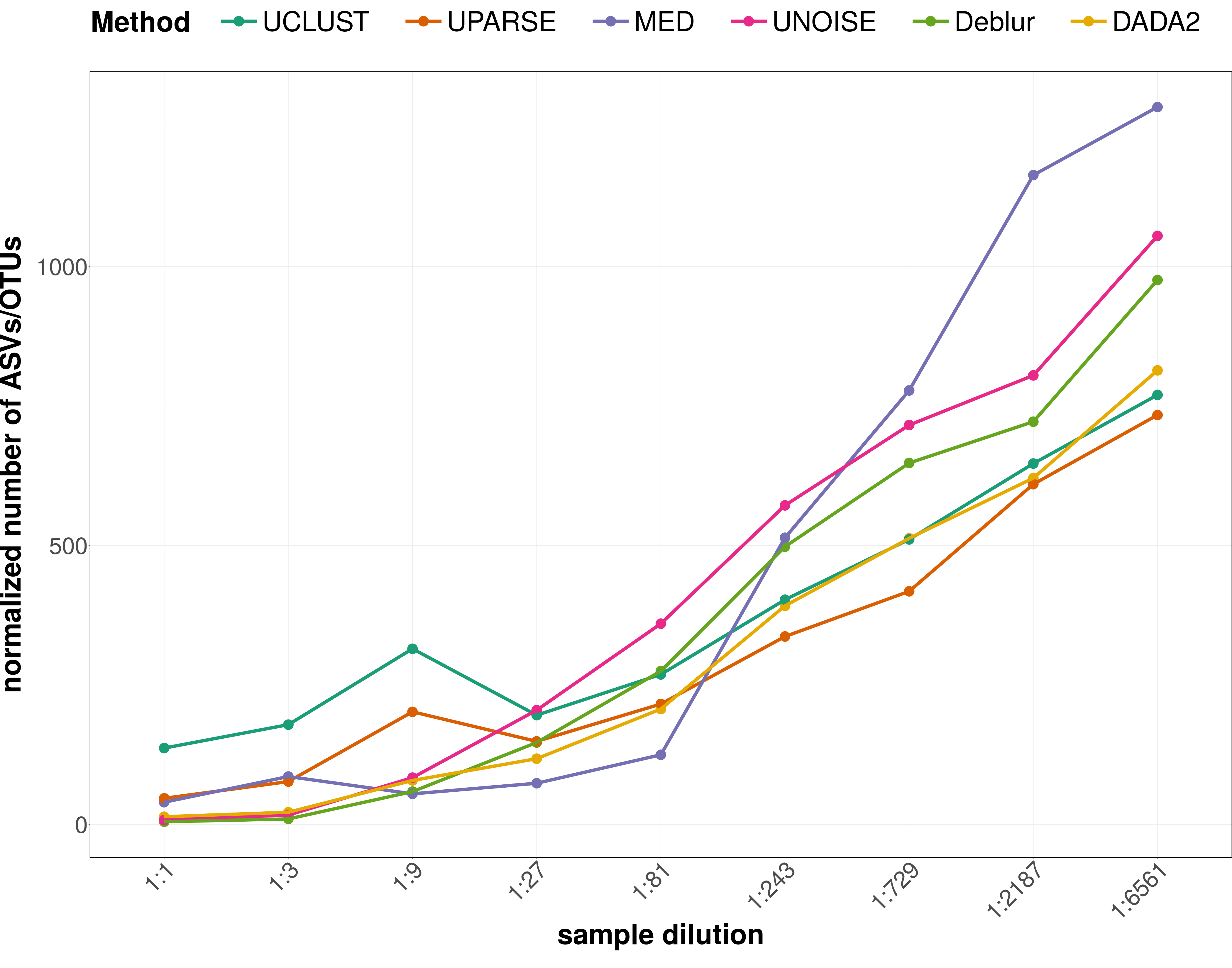

Supplement: FIG S2 [file mSystems.00163-18-sf002.tif]

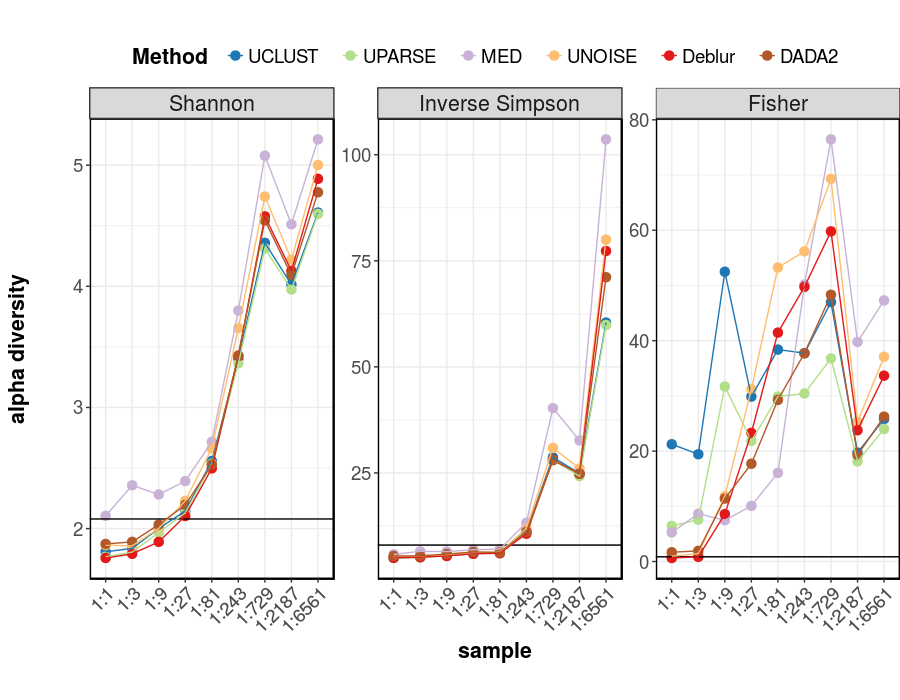

Supplement: FIG S3 [file mSystems.00163-18-sf003.tif]
